# Neuroendocrine and psychophysiological investigation of the evolutionary roots of gossip

**DOI:** 10.1038/s41598-023-30126-9

**Published:** 2023-02-22

**Authors:** Konrad Rudnicki, Irina Spacova, Charlotte De Backer, Caroline E. M. K. Dricot, Sarah Lebeer, Karolien Poels

**Affiliations:** 1 Department of Communication Science, University of Antwerp, Sint-Jacobstraat 2, 2000 Antwerp, Belgium; 2 Centre for Philosophical Psychology, University of Antwerp, Grote Kauwenberg 18, 2000 Antwerp, Belgium; 3 Department of Bioscience Engineering, University of Antwerp, Groenenborgerlaan 171, 2020 Antwerp, Belgium

**Keywords:** Human behaviour, Neurophysiology

## Abstract

This study investigates an evolutionary hypothesis of gossip postulating that in humans it serves a similar function as social grooming in other primates. It examines whether gossip decreases physiological markers of stress and increases markers of positive emotionality and sociability. Dyads of friends (N = 66) recruited at the university, participated in an experiment where they experienced a stressor followed by social interaction (gossip or control task). Individual levels of salivary cortisol and $\beta$-endorphins were assessed at before and after social interactions. Sympathetic activity and parasympathetic activity were monitored throughout the experiment. Individual differences in Tendency and Attitude towards Gossip were investigated as potential covariates. Gossip condition was characterized with increased sympathetic and parasympathetic activity, but did not differ in cortisol or $\beta$-endorphins levels. However, high Tendency to Gossip was associated with decreases in cortisol. Gossip was shown to be more emotionally salient than non-social talk, but the evidence with regard to lowering stress was not sufficient to support an analogy to social grooming.

## Introduction

Living in social groups provides substantial evolutionary advantages. Cooperation helps groups obtain resources and increases the survival chances of offspring, but it also entails many challenges that individuals have to face. In particular, social hierarchies expose individuals to stressors, such as harassment and exploitation by higher-ranking group members^1^. As a result, social animals had to develop mechanisms of building social cohesion and trust to maintain cooperation and alleviate the stress of group living. In most primates, social grooming is one of the main mechanisms responsible for that^2^. Primates spend as much as 20% of their waking time cleaning and gently touching each other, which is enough to maintain trust within groups as large as 50 individuals^2^. In comparison, humans naturally form social groups of up to 150 individuals, even though they spend significantly less time on social grooming than other primates^3^. This means that humans must have found other, more effective ways of building trust and social cohesion. One of the hypotheses addressing that issue was formulated in the 1990s by Robin^4–6^, who proposed that language and, in particular—gossip—replaced social grooming in humans as a primary way of bond formation. He reasoned that verbal communication is more effective than physical grooming because it can be addressed to multiple group members simultaneously, which explains the rise of group size from 50 in non-human primates to 150 in humans. Furthermore, gossip, understood as an exchange of information about absent third parties, seems like an excellent way of promoting in-group cooperation by disseminating social information about which group members are trustworthy and which are not^7^. Despite the popularity of that hypothesis among evolutionary psychologists and communication scholars^8–10^, there were virtually no studies that would examine if there is neuroendocrine or psychophysiological evidence for it. The aim of this study is to address that and determine if interpersonal gossip in humans elicits similar neuroendocrine and psychophysiological effects as social grooming elicits in other primates.

Social grooming has well-established effects on the physiology of primates^3^. In particular, it is highly efficient at reducing stress levels via several mechanisms^11^. Physical contact during grooming is registered as an *innocuous sensory activation*, which involves separate neural pathways from other types of sensory stimulation^12^. That activation causes the release of hormones that counteract the stress response. For instance, in Talapoin monkeys, grooming causes a significant increase in the levels of $\beta$-endorphins^13^. Endorphinsi.e., endogenous opioids) are primarily involved in regulating pain perception by acting as analgesics^14^. In humans, their psychological effect is often described as the “runner’s high,’ characterized by a sense of relaxation and well-being^14^. In primates, it is regarded as one of the primary mechanisms underlying the bonding effect of social grooming^6^. That claim is well-founded since pharmacologically blocking the receptors for endorphins in macaques was shown to reduce the motivation to engage in grooming^15^. Suppose the hypothesis of Robin Dunbar holds true and gossip gradually replaced grooming. In that case, it can be expected that humans will exhibit increased secretion of $\beta$-endorphins during gossip compared to other social interactions (Hypothesis 1).

Other hormones were also shown to change as a result of social grooming. In particular, cortisol levels decrease when primates groom each other. This effect has been demonstrated in several primate species, including male baboons^16^, rhesus monkeys^11^, barbary macaques^17^ and male chimpanzees^18^. Cortisol is one of the most widespread markers of stress^19^. Cortisol release is one of the components of the fight-or-flight response and mediates the adverse effects of stress both in primates and in humans. Thanks to social grooming, primates can down-regulate cortisol levels and prevent the debilitating effects of chronic stress. Humans can do that as well since research shows that interpersonal touch alleviates our levels of cortisol^20^. However, we do not know if gossip can also have a similar effect on cortisol levels. Only one published study has ever reported cortisol levels before and after gossip (in a relatively small sample = 22) and found no significant changes^21^. However, the participants in the study by^21^ were not required to be friends with each other. In this study, we want to investigate if gossip affects cortisol levels during interactions between people who are already close to each other to relate these results to primates who do not groom strangers but only members of their social group. We hypothesize that gossiping will cause a more substantial decrease in cortisol levels than other types of social interactions between friends (Hypothesis 2).

The decrease in stress levels due to social grooming is evident not only in neuroendocrine data but also in psychophysiological signals that measure autonomic activity. Both branches of the autonomic nervous system are crucial regulators of stress and social behavior^22^. Sympathetic nervous system activation is a part of the stress response and shifts attentional resources to search for threats instead of bonding opportunities^23^. In contrast, parasympathetic nervous system activation is higher when the environment is perceived as safe and promotes sociability and bonding^22^. This is true for humans as well as other primates. Social grooming was shown to be related to decreased sympathetic activity of pigtail macaques^24^ and increased parasympathetic activity of rhesus monkeys^25^. Similarly, in humans, positive social interactions were shown to increase parasympathetic activity^26–29^, while negative emotionality and social anxiety were associated with an increase in sympathetic activity^30–32^. Surprisingly, there is still no human data whether gossip affects their autonomic activity. In line with the evolutionary hypothesis of gossip by Robin^6^, we hypothesize that gossiping will result in a more substantial decrease in sympathetic activity Hypothesis 3a and a more substantial increase in parasympathetic activity Hypothesis 3b compared to other social interactions.

Hypothesizing that gossip can alleviate the neuroendocrine and psychophysiological markers of stress relies on the assumption that a gossiping individual experienced stress in the first place. After all, one of the most important functions of grooming in primates is consolation after stress^33^. Similarly, in humans physical contact after stressors was shown to reduce stress levels regardless of the stressor type^34^. An example of such a behavior can be seen in children who seek social support from their parents after suffering injuries during play. Receiving that social support was shown to prevent the adverse effects of social stress^35^. To hypothesize that gossip acts in humans the same way as grooming in primates entails that it should serve as a tool of social support. That hypothesis was initially pursued by^36^, who showed that nurses indeed treat gossip as means of obtaining social support. However, in contrast,^37^ did not find any evidence for gossip being a coping mechanism. In online surveys, she found that more conflict in the workplace resulted in more gossip and that gossip did not help with relieving the stress stemming from that conflict. On the contrary, employees who gossiped more also experienced more stress. As a result, most research in organizational science treats gossip as a stressor that was shown to be moderately related to higher occupational stress^38^. These mixed results stem from the fact that gossip may be perceived drastically different depending on the position of an individual within the group. For gossipers, it may be a tool for defending against others who break the group norms (see,^1^, while for gossipees it is a threat to their reputation^39^. Therefore, it is essential to note that gossip is hypothesized to serve as a tool of social support and stress relief for the gossipers themselves, only if they trust each other and if they experienced some stressors before gossiping. As a result, in this study, we will investigate dyads of friends who separately experience a stressor and then come together to interact.

## Methods

### Participants

Students ($N=66$, $M_{age}=21.01$, $SD=1.64$, male $=16.6\%$) from the University of Antwerp were recruited via emails and online advertisements. Participants registered in dyads and were instructed to only register with someone they consider their friend. There were no requirements for the length of the friendship; however upon registration, participants reported how long they knew each other prior to the experiment ($M_{months}=35.53$, $SD=43.54$).

### Design and procedure

**Figure 1:**
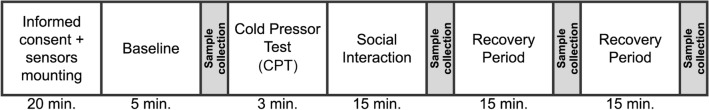
Timeline of the experiment.

A mixed 2x2 experimental design was used, where time serves as the within factor, while interaction type (gossip vs. control task) serves as the between factor. The timeline of the experiment is presented in Fig. 1.

The protocol of this study was approved by the Ethics Committee of the Antwerp University Hospital (Project ID 2021—1733—BUN B3002021000279). All procedures performed in this study were in accordance with the ethical standards of the institutional research committee and with the 1964 Helsinki declaration and its later amendments.

Upon arrival at the laboratory, participants were escorted to separate rooms and signed the informed consent forms. Next, two experimenters simultaneously prepared the participants by installing sensors for electrodermal activity (EDA) and electrocardiogram (ECG). After installing the sensors, participants were left alone in their respective rooms to collect a 5-min baseline recording. After the baseline recording, experimenters collected the first saliva sample for hormonal measurements. Samples were collected using the Salivettes$^{\copyright }$ (Sarstedt,Nümbrecht, Germany), which require participants to place cotton swabs in their mouth and chew for 60 s. The average saliva volume usually recovered from samples is $1.1 \pm 0.3$ ml according to the manufacturer.

Throughout the experiment, at four timepoints (baseline, after the stressor, after the social interaction and during recovery) participants filled out self-report questionnaires concerning their perceived emotional state (Physiological Arousal QuestionnaireSupplementary materials^40^; Self-Assessment Manikin^41^. The results of these analyses are included in the .

#### Stressor: the cold pressor test

After collecting the first samples, participants were subjected to the stressor—the Socially-Evaluated Cold Pressor Test (CPT)—the current golden standard for experimental stress elicitation and was consistently shown to cause an increase in cortisol levels^42^. In that task participants submerge their hands in a tank of cold water (0-2$^\circ C$) for as long as they can (up to 3 min). Additionally, during the submersion, participants are instructed to look into a camera recording their faces with the live recording playing in their field of vision. While the participants’ hands are in the cold water, the experimenter pretends to take notes and evaluate the participant, simulating the stressor of social evaluation. The participant is not allowed to talk during the CPT, and should they stop looking into the camera, the experimenter asks the participant to look into the camera again.

#### Social interaction: gossip task and control task

After finishing the CPT, participants were moved to the same room to engage in social interactions. Dyads of participants were randomly assigned to one of two conditions: gossip condition or control condition. In the gossip condition, participants received five vignettes describing various stories of controversial behavior by other people (e.g., cheating on a romantic partner, avoiding responsibilities at work/school). Each story was accompanied by a set of questions asking the participants to discuss whether something like that ever happened to them or to people they personally know and what they think about the behavior of those people. Participants were instructed to familiarize themselves with the vignettes and discuss the questions together for 15 min.

To examine if gossip is more efficient at stress relief than other types of interactions, we designed a control condition that would entail a discussion that does not involve any exchange of social information about absent people. In the control condition, participants received twenty pictures of common household objects and were instructed to devise creative ways of using them. Previous research already showed that this task does not elicit any social information exchange^43^.

After 15 min of social interaction, participants were moved to separate rooms, and the second round of saliva samples was collected. The following two saliva sample collections occurred 15 and 30 min after the end of the social interaction. Except for a short period when experimenters collected the third round of saliva samples, participants remained in solitude (recovery period) for 30 min following the social interaction. After the recovery period concluded participants were debriefed about the purpose of the experiment and remunerated with a fee of 30 euro per person.

### Psychophysiological recordings

To measure sympathetic activity, tonic and phasic components had to be derived from electrodermal activity (EDA). EDA was measured using a Shimmer3 GSR wearable device (Consensys) from electrodes placed on participants’ palms (sampling rate: 128 Hz). EDA signal was decomposed into two sub-components: tonic and phasic EDA. Both measures were obtained using continuous decomposition analysis with Ledalab software following the procedures devised by^44^. Tonic EDA indicates a slowly-changing general level of arousal and sympathetic activity^45^, while phasic EDA signifies rapid sympathetic reactivity to environmental stimuli^46^.

To measure parasympathetic activity, heart rate variability (HRV) had to be derived from an electrocardiogram (ECG). ECG was recorded with Shimmer3 ECG wearable device (Consensys) from electrodes placed on participants’ chests (sampling rate: 512 Hz). To derive HRV, R-peaks were detected using Kubios HRV software (Biomedical Signal Analysis Group, Department of Applied Physics University of Kuopio, Finland). The Fourier transform was used to obtain power spectra of the inter-beat-interval signal waveform, and the log-transformed high-frequency band (0.15-0.4 Hz) was extracted as a marker of parasympathetic activation^47^.

High-frequency HRV was selected for this study, since it is the most widely used marker of parasympathetic activation in particular, and is not influenced by sympathetic innervation of the heart^47^. As a result, it is positively correlated with positive emotions during social interactions (e.g, cheerfulness^29^ and negatively correlated with stress and anxiety^47^.

Psychophysiological measurements have a much higher temporal resolution than hormonal measurements since it takes several minutes for hormones to gradually excrete from the organism. As a result, within the time of the experiment, we were able to collect four saliva samples. In contrast, EDA can be measured continuously at 128Hz, while HF-HRV is recommended to be averaged over periods of approximately 5 min^47^. Therefore, EDA and HF-HRV were averaged over 5-min periods throughout the whole experiment, with the exception of CPT, which lasted 3 min.

### Hormone levels

All of the saliva samples were immediately stored at -20°C upon collection. All of the samples were analyzed within 3 months from the collection. After thawing, Salivettes ^©^ were centrifuged for 2 min at 1000$\times$*g*.

Salivary cortisol levels were assessed using the enzyme-linked immunosorbent assay kit (ELISA) by Enzo Life Sciences (ADI-900-071) according to the manufacturer instructions. Inter- and intra-assay variation coefficients were $6.7\%$ and $3.2\%$ respectively.

Salivary $\beta$-endorphins levels were assessed using the ELISA kit by Novus Biologicals (NBP2-78774) according to the manufacturer instructions. Inter- and intra-assay variation coefficients were $13.6\%$ and $6.5\%$ respectively.

Samples were registered at the *Biobank Antwerpen*, Antwerp, Belgium; ID: BE 71030031000 (528 samples—human saliva)^48^.

Because it takes time for cortisol and $\beta$-endorphins to permeate from organs into saliva, the collections of saliva samples had to be planned with a time delay between the tasks and the target samples. Past research on stress, cortisol and $\beta$-endorphins allowed between 15 and 45 min for the analytes to permeate into saliva^49,50^. In this study, the sample collected right after the social interaction represents hormone levels affected by the Cold Pressor Test, and the samples collected during the recovery period represent hormone levels affected by the social interaction.

Cortisol exhibits high diurnal variation—its levels surge after waking, rapidly decrease for the next few hours and then decline slowly until nighttime^51^. To reduce the impact of that variance on the results of this study, all of the cortisol collections took place after 12PM local time.

### Individual differences in gossip

Past research revealed that people differ in their attitudes towards gossip and tendency to engage in gossip^52^. Those differences were shown to moderate the outcomes of social interactions^53,54^. In order to ensure that the effects of gossip on hormone levels and psychophysiology are not dependent on individual differences, participants filled out two questionnaires upon registration.

To estimate how often do participants engage in gossip spontaneously, they filled out the Tendency to Gossip Questionnaire developed by Nevo et al.^55^ The scale consists of 20 items rated on a 7-point scale (from *never* to *always*) which form 4 subscales describing tendency to gossip about different topics (*appearance, social information, achievement, sublimated*)(Cronbach $\alpha = 0.82$). Then, to assess participants’ opinions about the morality of gossip, they completed the Attitudes Towards Gossip scale created by^52^. It consists of 29 items rated on a 5-point scale (from *disagree strongly* to *agree strongly*) (Cronbach $\alpha = 0.80$).

## Results

In order to examine if hormone levels, autonomic activity and self-reported perceptions changed as a function of time, repeated measures ANOVAs were performed with the type of social interaction (gossip vs. control) set as the between-subjects factor and individual differences (Tendency to Gossip, Attitude Towards Gossip) set as covariates. Greenhouse-Geisser corrections were applied in the models where the assumption of variance-covariance matrix sphericity was violated. Analyses of normality revealed that the distributions of cortisol levels and $\beta$-endorphins levels were positively skewed. To satisfy the assumptions of the performed statistical tests hormone levels were log-transformed prior to the analysis.

To investigate whether the length of friendship affected the effects of social interactions on hormone levels and psychophysiological recordings, it was included as a co-variate in all the analyses. The results revealed no effect of the length of friendship on interaction-induced changes in any of the study variables. The only effect of the friendship length was visible during CPT. Participants’ who knew each other longer exhibited higher cortisol release during CPT $F(2.5, 135.43) = 2.99, p < 0.05, \eta ^2 = 0.05$.

### $\beta$-endorphins

**Figure 2:**
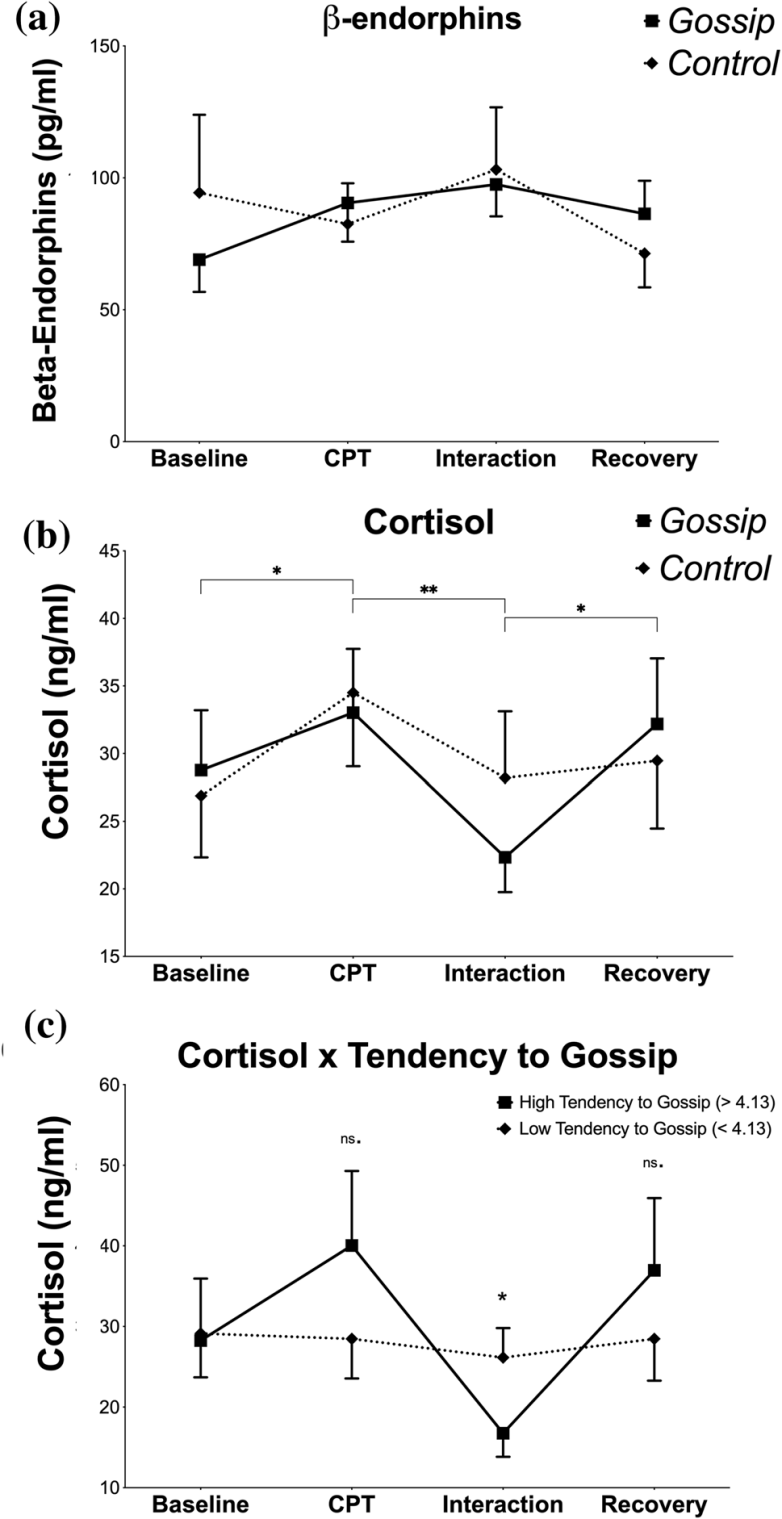
Changes in hormone levels during the experiment. (**a**) changes in cortisol depending on the type of social interaction (gossip vs. control), (**b**) changes in cortisol in the gossip condition depending on the Tendency to Gossip, (**c**) changes in $\beta$-endorphins depending on the type of social interaction (gossip vs. control). *$p < 0.05,$ **$p < 0.01$, ns. - insignificant, error bars: ± SEM. Horizontal brackets indicate which time-points significantly differed from each other. Asterisks without the brackets indicate that at that timepoint gossip condition significantly differed from the control condition.

Changes in $\beta$-endorphins levels are presented in Fig. 2c.

$\beta$-endorphins levels did not significantly change as a result of CPT, $F(1.02, 45) = 2.43, p = 0.13, \eta ^2 = 0.05$, but significantly increased as a result of the social interaction, $F(1, 45) = 4.52, p < 0.05, \eta ^2 = 0.09$ and decreased during recovery $F(1, 46) = 14.97, p < 0.05, \eta ^2 = 0.25$. There were no significant differences between the gossip condition and the control condition in the levels of $\beta$-endorphins at any point $F(1.90, 81.47) = 1.37, p = 0.26, \eta ^2 = 0.03$.

Neither Tendency to Gossip, $F(1.66, 33.18) = 2.09, p = 0.15, \eta ^2 = 0.10$, nor Attitude Towards Gossip, $F(1.66, 33.18) = 0.55, p = 0.55, \eta ^2 = 0.03$, were significant covariates of the levels of $\beta$-endorphins.

$\beta$-endorphins levels showed several correlations to other neuroendocrine and psychophysiological measurements (see Supplementary materials).

Statistical analysis of $\beta$-endorphins found no evidence for the hypothesis 1 that gossip alleviates stress through increasing $\beta$-endorphins secretion.

### Cortisol

Changes in cortisol levels are presented in Fig. 2a.

Cortisol levels significantly rose as a result of CPT, $F(1, 62) = 8.78, p < 0.01, \eta ^2 = 0.12$, and significantly decreased as a result of the social interaction, $F(1, 61) = 6.74, p < 0.05, \eta ^2 = 0.10$. There were no significant differences between the gossip condition and the control condition in the rate of that decrease $F(1, 61) = 0.00, p = 0.99, \eta ^2 = 0.00$.

However, Tendency to Gossip significantly covaried with the effects of gossiping on cortisol decrease, $F(1, 30) = 5.40, p < 0.05, \eta ^2 = 0.15$, while Attitude Towards Gossip did not $F(1, 29) = 1.04, p = 0.32, \eta ^2 = 0.03$. The interaction between the Tendency to Gossip and cortisol levels is presented in Fig. 2b.

Statistical analysis of salivary cortisol levels found partial support for the hypothesis 2 that gossip alleviates stress through reducing cortisol secretion. The hypothesized effects seems to be present only in people with high Tendency to Gossip.

### Sympathetic activity

**Figure 3:**
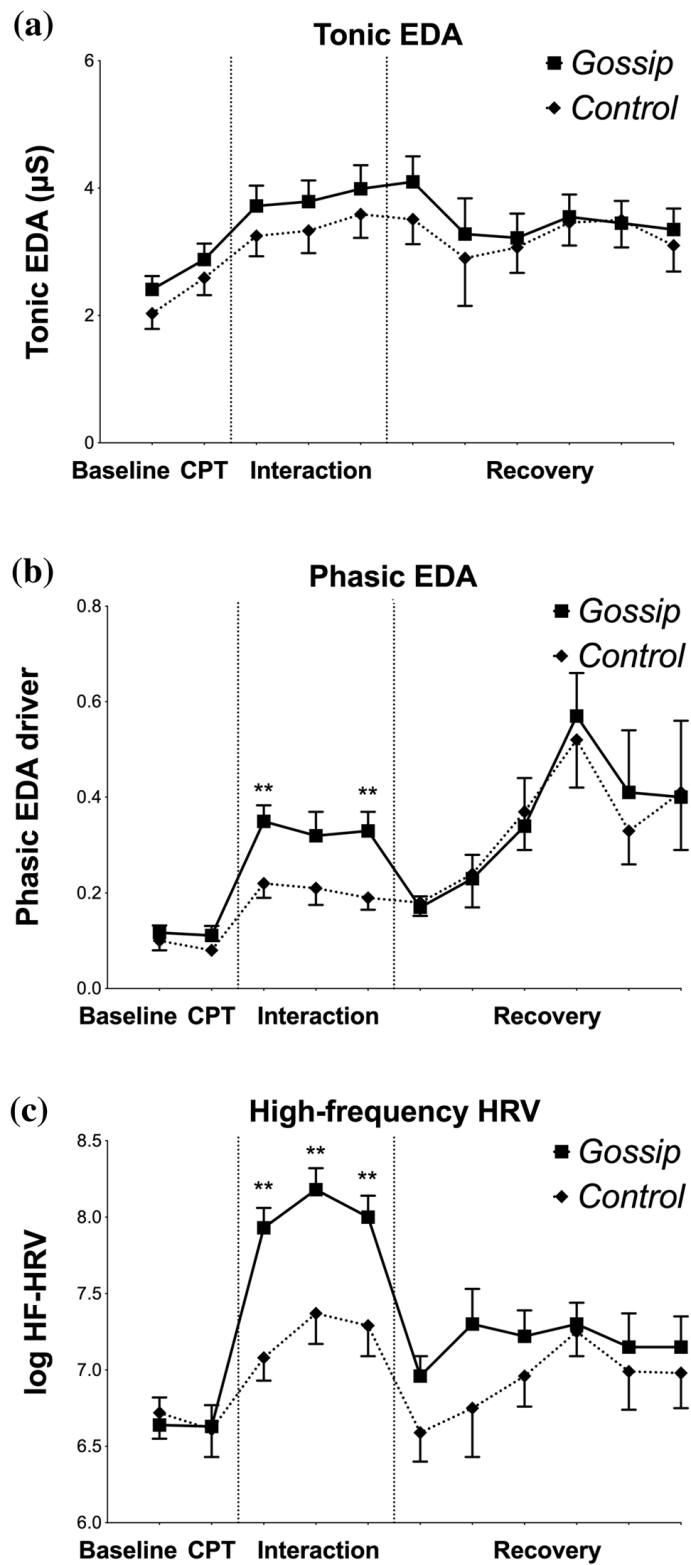
Changes in participants’ autonomic activity during the experiment. Vertical dotted lines mark the beginning and end of the social interaction. Each datapoint represents a 5-min epoch, with the exception of CPT, which lasted 3 min. EDA— Electrodermal Activity, CPT-Cold Pressor Test, HF-HRV—High Frequency Heart Rate Variability. *$p < 0.05,$ **$p < 0.01$, error bars: ± SEM. Asterisks without the brackets indicate that at that timepoint gossip condition significantly differed from the control condition.

The changes in sympathetic activity are presented in Fig. 3a,b.

Endogenous, slowly changing level of arousal measured with tonic EDA significantly increased from baseline to CPT and throughout the social interaction, $F(1.32, 84.36) = 27.12, p < 0.01, \eta ^2 = 0.30$, but these increases did not differ between gossip and control conditions, $F(1,32, 84.36) = 27.12, p = 0.84, \eta ^2 = 0.00$. Changes in tonic EDA were not affected by neither Attitude Towards Gossip, $F(1.36, 40.67) = 0.83, p = 0.51, \eta ^2 = 0.03$, nor Tendency to Gossip, $F(1.36, 40.67) = 0.82, p = 0.41, \eta ^2 = 0.03$.

Arousal in response to environmental stimuli measured with phasic EDA did not change as a result of CPT, $F(1, 64) = 0.62, p = 0.44, \eta ^2 = 0.01$, but significantly changed as a result of the social interaction, $F(2.43, 155.9) = 29.70, p<0.01, \eta ^2 = 0.32$, and these changes differed between gossip and control conditions, with gossip condition eliciting higher phasic EDA, $F(2.20, 140.91) = 4.22, p < 0.05, \eta ^2 = 0.06$.

**Figure 4:**
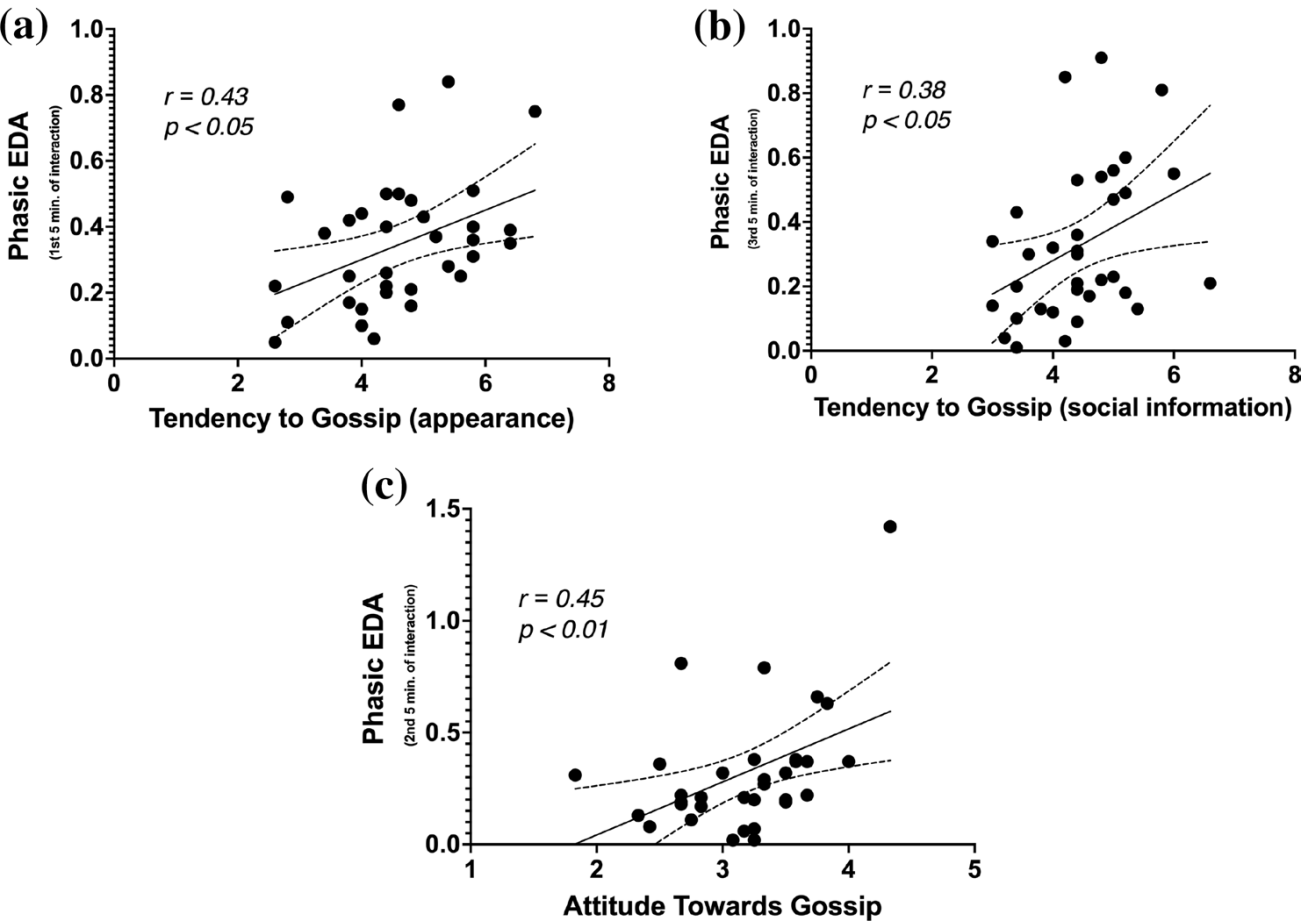
Correlations between Phasic EDA levels during the social interaction in the gossip condition and individual differences with regard to gossip. EDA-Electrodermal Activity. Dotted lines represent $95\%$ confidence intervals.

The effects of gossip on phasic EDA were significantly affected by Attitude Towards Gossip, $F(2.58, 77.28) = 3.00, p < 0.05, \eta ^2 = 0.09$, but not Tendency to Gossip, $F(2.58, 77.28) = 0.38, p = 0.82, \eta ^2 = 0.01$. Participants with more positive Attitude Towards Gossip exhibited stronger phasic EDA increase. Consistently, both Tendency to Gossip and Attitude Towards Gossip positively correlated with phasic EDA (see Fig. 4).

Statistical analysis of phasic and tonic EDA found no support for the hypothesis 3a that gossip alleviates stress through reducing sympathetic activation. On the contrary, gossip caused higher phasic EDA levels than the control task, especially in people with higher attitude towards gossip.

### Parasympathetic activity

The changes in parasympathetic activity are presented in Fig. 3c.

Parasympathetic activity measured with HRV did not change as a result of CPT, $F(1, 60) = 0.30, p = 0.59, \eta ^2 = 0.01$, but significantly increased as a result of the social interaction $F(2.75, 161.97) = 28.34, p < 0.01, \eta ^2 = 0.32$. HRV increased significantly more in the gossip condition than in the control condition $F(2.75, 161.97) = 4.53, p < 0.01, \eta ^2 = 0.07$.

The effects of gossip on HRV were not affected by neither Attitude Towards Gossip, $F(2.78, 83.451) = 1.97, p = 0.10, \eta ^2 = 0.06$, nor Tendency to Gossip, $F(2.78, 83.451) = 1.84, p = 0.15, \eta ^2 = 0.06$.

Statistical analysis of HRV found evidence for the hypothesis 3b that gossip promotes sociability through enhancing parasympathetic activation.

## Discussion

This study aimed to investigate whether gossip between human friends can be considered a bonding mechanism akin to social grooming in other primates. To answer that question, we performed an experiment in which neuroendocrine and psychophysiological markers that mediate the bonding effects of social grooming in primates were monitored in gossiping humans. We hypothesized that gossip will increase parasympathetic activity and salivary levels of $\beta$-endorphins, while decreasing sympathetic activity and salivary levels of cortisol. The results showed that: a) gossip increases both sympathetic and parasympathetic activity, b) does not alter $\beta$-endorphin levels, and c) can only conditionally lower cortisol levels. Taken together, these results lend themselves to the idea that gossip is a salient emotional cue, which affects autonomic activity during social interactions^56^. However, the evolutionary hypothesis that gossip in humans is an equivalent of social grooming in other primates remains unresolved^6^.

In this study, gossip did not affect salivary $\beta$-endorphins. This suggests that the neuroendocrine mechanism responsible for bonding in primates during social grooming^57^ is not active when humans gossip after physical stressors. Salivary $\beta$-endorphins in humans can be interpreted in two ways. First, their levels are strongly correlated with feelings of euphoria, which manifests itself, for example, when people jump on a bungee^50^ or take psychoactive substances^58^. Most commonly, those positive feelings are called *the runner’s high* because $\beta$-endorphins are responsible for the euphoric effects of physical exercise in humans^59–61^. Second, $\beta$-endorphins are sometimes used as stress markers^62^. These two seemingly contradictory interpretations stem from the same underlying function of $\beta$-endorphins. Namely, as endogenous opioids, they are responsible for alleviating pain when necessary and for facilitating the activity of the reward system^63,64^. As a result, when people engage in risky behaviors (e.g., bungee jumping or drug use) or enter stressful situations, the endogenous opioid system anticipates possible pain and releases analgesic $\beta$-endorphins. Indeed, as markers of stress, $\beta$-endorphins are used among stomatology researchers interested in preventing dental patients’ pain^65,66^. The relaxing, analgesic and euphoric properties of $\beta$-endorphins may be responsible for mediating the effects of social grooming on bonding in non-human primates^57^. However, more research is required to test whether that is true also in humans. Gossip surely serves a role in human bonding, as evidenced by other studies^43,67^, but the patterns of $\beta$-endorphins release after a socially evaluated physical stressor did not differ as a function of gossip.

In contrast to $\beta$-endorphin, when cortisol is used as a biomarker in psychological research, it is almost exclusively an indicator of acute stress^68^. In this experiment, to elicit cortisol secretion, we employed one of the most reliable experimental manipulations of stress—the Cold Pressor Test (CPT)^69^. Recent meta-analyses confirmed its effectiveness^69^ and consistently, in our experiment, CPT caused an increase in cortisol levels. Thus, we were able to observe if gossip would be more effective at reducing cortisol compared to an interaction that did not involve talking about other people. The results showed that it was not, as cortisol levels in the gossip condition did not significantly differ from the control condition. This is consistent with the study by^21^, who found no effect of gossip on cortisol levels. There are many positive interactions between humans that were shown to help with stress by decreasing cortisol levels. Cortisol drops when we are hugged^70^, listen to someone read us a story^71^ or listen to music^72^. The evolutionary hypothesis of gossip predicts that gossip should act similarly to those interactions. Even though in our study gossip did not elicit greater changes in cortisol than the control task, it could be partly due to the nature of the stressor. Cold Pressor Test involves both physical pain and social evaluation, while the evolutionary hypothesis of gossip focuses solely on social stressors. Future studies should address this limitation and investigate whether gossip exhibits a stronger influence over social stressors (such as the Trier Social Stress Test). These future studies are critical since our study did find some link between gossip and cortisol release. Namely, we found that the tendency to gossip moderates the effects of gossip on cortisol levels^55^. Participants who reported higher tendency to gossip exhibited a significant reduction in cortisol levels during the gossip task (see Fig. 2b). The fact that gossip was effective enough at alleviating stress only in people who frequently gossip may suggest that it is an effective coping mechanism only when it constitutes a valuable tool for the gossipers. In other words, gossip might help with stress only when the gossiper sees a clear purpose for their gossip (e.g., status gain, manipulating the reputation of others) (see^73^. Because gossip is used to improve social status, those with a high tendency to gossip are typically those who lack that status in the first place^74^. Future work should consider whether gossip is an effective coping mechanism for those who rank low within dominance hierarchies and lack social status^73^.

To examine if gossip facilitates sociability and positive emotionality, this study also examined moment-to-moment changes in sympathetic activity (i.e., arousal) measured with electrodermal activity (EDA). The results showed that phasic EDA—a measure of rapid, stimuli-driven arousal spikes—was significantly higher in the gossip condition than in the control condition. This result is contrary to the original hypothesis that gossip alleviates arousal. This counter-intuitive result likely stems from a misinterpretation of the role of the sympathetic nervous system in an interaction between friends (i.e., a safe, non-threatening stimulus). In most research, EDA is used as a marker of stress or *negative* arousal^75^. However, sympathetic nervous system produces arousal not only as a part of the *fight-or-flight* response but as a part of many positive emotions as well^76,77^. In other words, EDA can only be used to measure the *arousal* aspect of the emotional response, while the *valence* aspect has to be determined otherwise. As a result, it was not possible to determine whether the arousal during the studied social interactions was experienced as positive or negative. Social cues are generally known to be very salient and elicit arousal, which may explain the results obtained in this study^78,79^. Consistently, we also discovered that people who self-reported a higher tendency to gossip and a more positive attitude towards gossip exhibited stronger phasic EDA responses during the gossip task (see Fig. 4c). This shows that the saliency of gossip is reflected in the magnitude of sympathetic response when it happens. Interestingly, the same can be said about the relation between gossip and parasympathetic response.

Parasympathetic nervous system activation is thought to promote social interactions by diminishing the *fight-or-flight* response^80^. In the absence of threats, parasympathetic activation increases and shifts motivation to socialization. Consequently, high-frequency heart rate variability (HRV), which is a marker of parasympathetic activation, has been shown to positively correlate with social approach^27,57^, promote cooperation^81^ and social cognition^82^. Because feelings of safety are a prerequisite for parasympathetic activation, HRV is also positively related to emotions with positive valence, like calmness and cheerfulness^28^. In our experiment, HRV increased significantly more in the gossip condition, suggesting that in that condition participants’ motivational resources were directed more towards social cues and that the valence of emotions they experienced was more likely to be positive. Together with an increase in sympathetic activity, these results indicate that gossip is significantly different at eliciting emotional responses from other, regular interactions between friends.

One of the limitations of the current study is the absence of a manipulation check. In particular, we were unable to record the participants’ conversations to ensure beyond any doubt that they did not gossip in the control condition and that they did in the experimental condition. However, not recording the participants was a conscious decision intended to increase the ecological validity of the results. We know people change their behavior unpredictably when observed^83^. Usually, this is already an issue for the validity of the collected data, but it becomes especially problematic when sensitive data is being collected. We asked participants to discuss the controversial behavior of people they know. Recording their conversations would raise two dire problems. First, it would be an ethical violation with regard to the personal data of the people our participants would discuss. Second, since participants would be aware of being recorded—the content of their gossip would be less likely to contain sensitive information. This would reduce the ecological validity of the experimental manipulation since it is precisely the sensitive nature of the exchanged social information that is hypothesized to be responsible for the effects of gossip.

Another potential limitation of this study is the skewed proportion of women to men in the analyzed sample. There are two important sex differences relevant to studying the influence of gossip on stress. First, men and women differ in their likelihood of engaging in gossip and their motivations to do so^84,85^. Men were shown to use gossip to improve their access to resources by learning new information^85^. In contrast, women gossip more to bond and manipulate reputation^84^. In particular, gossip is important for women when resolving conflicts^86^. The second sex difference concerns reactivity to stress. Men and women are known to react to different stressors with different magnitudes, but most importantly, they also engage in different behaviors to cope with them^87^. The results of this study pertain primarily to women since they constituted the majority of the participants. Future research should address whether gossip has different effects on stress in men and women.

Overall, this study found no evidence supporting the evolutionary hypothesis that gossip in humans is analogous to social grooming in other primates. However, the results have not strongly contradicted that hypothesis either. $\beta$-endorphins did not change as a result of social interactions, while cortisol level decreases were contingent on individual differences in the tendency to gossip. The results also revealed that gossip differs from a joint creativity task between friends by resulting in increased arousal and sociability. This result supports the notion that gossip facilitates in-group cooperation by constituting a salient channel of social information exchange.

## Supplementary Information

Supplementary Information.

## Data Availability

The datasets used and/or analysed during the current study available from the corresponding author on reasonable request.
