# Supplementary material for: Neuroendocrine and psychophysiological investigation of the evolutionary roots of gossip
Source: Sci Rep. 2023 Feb 22;13:3117. doi: 10.1038/s41598-023-30126-9 (PMC9946955; doi:10.1038/s41598-023-30126-9)
Supplement: Supplementary file 1 — Supplementary Information. [file 41598_2023_30126_MOESM1_ESM.pdf]

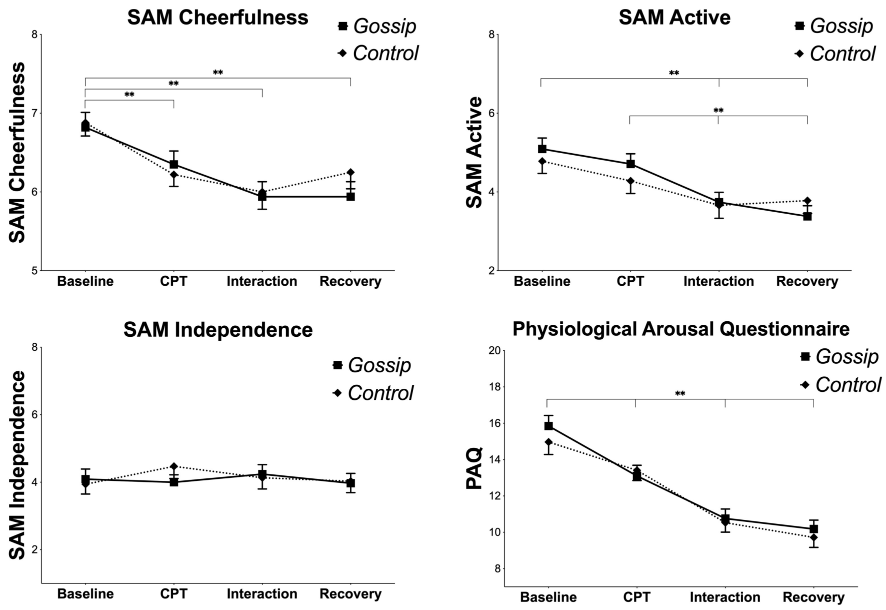

Fig. 1 Changes in participants' self reported emotions and perceived stress during the experiment. SAM - Self-Assessment Manikin, CPT - Cold Pressor Test, $*-p < 0.05$, $**-p < 0.01$. Horizontal brackets indicate which time-points significantly differed from each other.

1 Supplementary material - Descriptive statistics, correlations and self-report results.

1.1 Self-report

The changes in self-reported measures are presented in Figure 1.

The results showed that the participants perception of *cheerfulness* significantly decreased throughout the experiment, $F(2.63, 168.34) = 14.61, p < 0.01, \eta^2 = 0.19$, but did not differ between the gossip and control condition, $F(2.63, 168.34) = 0.8, p = 0.50, \eta^2 = 0.01$.

The participants' perception of how *active* they felt significantly decreased throughout the experiment, $F(2.62, 167.57) = 19.85, p < 0.01, \eta^2 = 0.24$, but did not differ between the gossip and control condition, $F(2.62, 167.57) = 1.57, p = 0.20, \eta^2 = 0.02$.

The participants' perception of how *independent* they felt has not changed throughout the experiment, $F(2.58, 164.79) = 1.03, p = 0.38, \eta^2 = 0.02$, and did not differ between the gossip and control condition, $F(2.58, 164.79) = 1.48, p = 0.22, \eta^2 = 0.02$.

The participants' perceived arousal has significantly decreased throughout the experiment, $F(2.20, 140.91) = 69.68, p < 0.01, \eta^2 = 0.52$, but did not differ between the gossip and control condition, $F(2.20, 140.91) = 0.70, p = 0.56, \eta^2 = 0.01$.

---

[0]Values = Mean ± SD. CPT - Cold Pressor Test, PAQ - Physiological Arousal Questionnaire.

Table 1 Descriptive statistics - psychophysiological signals

Time	Gossip			Control		
	Phasic EDA	Tonic EDA	HF-HRV$_{log}$	Phasic EDA	Tonic EDA	HF-HRV$_{log}$
Baseline	0.12 ± 0.09	2.42 ± 1.25	6.65 ± 1.05	0.11 ± 0.10	2.03 ± 1.39	6.72 ± 0.99
CPT	0.11 ± 0.11	2.88 ± 1.50	6.63 ± 0.79	0.09 ± 0.08	2.60 ± 1.53	6.61 ± 0.98
Interaction (1_{st} 5 min.)	0.34 ± 0.19	3.73 ± 1.87	7.94 ± 0.76	0.21 ± 0.18	3.26 ± 1.85	7.09 ± 0.77
Interaction (2_{nd} 5 min.)	0.32 ± 0.28	3.80 ± 1.94	8.18 ± 0.81	0.20 ± 0.19	3.34 ± 2.03	7.37 ± 1.08
Interaction (3_{rd} 5 min.)	0.32 ± 0.24	4.00 ± 2.18	7.99 ± 0.84	0.19 ± 0.15	3.60 ± 2.09	7.29 ± 1.07
Recovery (1_{st} 5 min.)	0.17 ± 0.14	4.10 ± 2.35	6.97 ± 0.76	0.18 ± 0.15	3.52 ± 2.02	6.59 ± 1.04
Recovery (2_{nd} 5 min.)	0.24 ± 0.26	3.29 ± 2.34	7.31 ± 1.21	0.23 ± 0.20	2.93 ± 2.84	6.75 ± 1.48
Recovery (3_{rd} 5 min.)	0.34 ± 0.54	3.23 ± 2.07	7.22 ± 0.84	0.37 ± 0.42	2.93 ± 2.84	6.97 ± 0.95
Recovery (4_{th} 5 min.)	0.57 ± 0.50	3.55 ± 1.99	7.30 ± 0.71	0.53 ± 0.56	3.46 ± 2.02	7.25 ± 0.79
Recovery (5_{th} 5 min.)	0.42 ± 0.75	3.46 ± 1.97	7.15 ± 1.08	0.33 ± 0.41	3.51 ± 2.36	7.00 ± 1.21
Recovery (6_{th} 5 min.)	0.40 ± 0.79	3.36 ± 1.70	7.15 ± 0.91	0.41 ± 0.61	3.11 ± 2.05	6.98 ± 1.05

Values = Mean ± SD. CPT - Cold Pressor Test.

Table 2 Descriptive statistics - Self-Assessment Manikin

	Gossip			Control		
Time	SAM Independent	SAM Active	SAM Cheerful	SAM Independent	SAM Active	SAM Cheerful
Baseline	4.09 ± 1.75	5.09 ± 1.64	6.82 ± 1.09	3.94 ± 1.63	4.78 ± 1.77	6.88 ± 0.94
CPT	4.00 ± 1.28	4.71 ± 1.53	6.35 ± 0.98	4.47 ± 2.02	4.28 ± 1.82	6.22 ± 0.87
Interaction	4.24 ± 1.62	3.74 ± 1.44	5.94 ± 1.10	4.13 ± 1.88	3.66 ± 1.84	6.00 ± 1.24
Recovery	3.38 ± 1.56	3.80 ± 1.94	5.94 ± 1.10	4.03 ± 1.93	3.78 ± 1.85	6.25 ± 1.19

Values = Mean $\pm$ SD. CPT - Cold Pressor Test, SAM - Self Assessment Manikin.

Table 3 Descriptive statistics - hormones and perceived arousal

	Gossip			Control		
Time	Cortisol (ng/ml)	β-endorphins (pg/ml)	PAQ	Cortisol (ng/ml)	β-endorphins (pg/ml)	PAQ
Baseline	28.77 ± 4.43	68.99 ± 12.22	15.85 ± 3.39	26.88 ± 4.55	94.39 ± 29.51	14.97 ± 3.93
CPT	33.02 ± 4.73	90.52 ± 14.71	13.09 ± 3.46	34.50 ± 5.44	82.47 ± 15.47	13.41 ± 3.19
Interaction	22.34 ± 2.58	97.49 ± 12.07	10.76 ± 3.00	28.19 ± 4.93	103.15 ± 23.61	10.53 ± 2.93
Recovery	32.18 ± 4.86	86.37 ± 12.55	10.18 ± 2.85	29.46 ± 4.99	71.33 ± 12.84	9.72 ± 3.13

PAQ - Physiological Arousal Questionnaire

Table 4 Correlations between the hormone levels, psychophysiological recordings and perceived arousal - baseline

-	Cortisol_{log}	β -endorphins_{log}	HF-HRV_{log}	Phasic EDA	Tonic EDA	PAQ
Cortisol_{log}	-	-	-	-	-	-
β -endorphins_{log}	0.28	-	-	-	-	-
HF-HRV_{log}	0.17	0.14	-	-	-	-
Phasic EDA	0.02	−0.17	0.11	-	-	-
Tonic EDA	−0.07	−0.11	0.01	0.65**	-	-
PAQ	0.07	0.04	0.07	0.23	0.31*	-

* - $p < 0.05$, ** - $p < 0.01$. PAQ - Physiological Arousal Questionnaire

Table 5 Correlations between the hormone levels, psychophysiological recordings and perceived arousal - CPT

-	$Cortisol_{log}$	β -endorphins$_{log}$	HF-HRV$_{log}$	Phasic EDA	Tonic EDA	PAQ
$Cortisol_{log}$	-	-	-	-	-	-
β -endorphins$_{log}$	0.21	-	-	-	-	-
HF-HRV$_{log}$	0.08	0.06	-	-	-	-
Phasic EDA	-0.07	-0.31^{*}	0.04	-	-	-
Tonic EDA	-0.06	-0.31^{*}	0.04	0.58^{**}	-	-
PAQ	0.18	0.02	-0.02	0.03	-0.04	-

* - p< 0.05, ** - p< 0.01. PAQ - Physiological Arousal Questionnaire, CPT - Cold Pressor Test

Table 6 Correlations between the hormone levels, psychophysiological recordings and perceived arousal - social interaction

-	$Cortisol_{log}$	β -$endorphins_{log}$	HF-HRV_{log}	Phasic EDA	Tonic EDA	PAQ
$Cortisol_{log}$	-	-	-	-	-	-
β -$endorphins_{log}$	0.32*	-	-	-	-	-
HF-HRV_{log}	0.10	−0.23	-	-	-	-
Phasic EDA	−0.05	0.00	0.19	-	-	-
Tonic EDA	0.17	−0.01	−0.07	0.38**	-	-
PAQ	0.23	−0.03	0.19	−0.05	−0.01	-

* - $p < 0.05$, ** - $p < 0.01$. PAQ - Physiological Arousal Questionnaire. Values for tonic EDA, phasic EDA and HF-HRV were obtained by averaging over the entire time of the social interaction
